# Surgical management and outcome of synovial sarcoma in the spine

**DOI:** 10.1186/s12957-018-1471-x

**Published:** 2018-08-27

**Authors:** Minglei Yang, Nanzhe Zhong, Chenglong Zhao, Wei Xu, Shaohui He, Jian Zhao, Xinghai Yang, Jianru Xiao

**Affiliations:** Department of Orthopedic Oncology, Changzheng Hospital, Second Military Medical University, 415 Fengyang Road, Shanghai, 200003 China

**Keywords:** Synovial sarcoma, Spine, Surgery, Prognosis

## Abstract

**Background:**

Synovial sarcoma (SS) is a soft tissue sarcoma that rarely occurs in the spine, and a minimal number of cases have been reported in the literature. Spinal SS is challenging in diagnosis and treatment and has a poor prognosis. The aim of this study was to summarize and analyse the clinical features and outcomes of patients with spinal SS.

**Methods:**

A total of 16 cases of patients with spinal SS admitted to our institution were reviewed retrospectively. General information, radiological findings and treatment strategies were collected. These patients were followed up regarding their continuing treatment, local or distant recurrence and survival.

**Results:**

Spinal SS patients in this series ranged in age from 12 to 68 years (median, 33). Four en bloc resections and 12 piecemeal resections were performed. Improved Frankel (*P* = 0.002), visual analogue scale (*P* = 0.002) and Karnofsky Performance Status (*P* = 0.002) scores were seen postoperatively. The mean follow-up period was 35.9 ± 23.5 (median 31.5, range 4–87) months, with four local recurrences and three distant metastases detected. Eight patients (50.0%) died of disease by the last follow-up. The 1-, 3- and 5-year overall survival rates were 87.5%, 61.4% and 40.9%, respectively. Preoperative chemotherapy was used in three patients to facilitate surgical resection, and adjuvant chemotherapy and radiotherapy were used in six patients.

**Conclusions:**

Spinal SS has a relatively high risk of local recurrence and distant metastasis. Surgical intervention can improve the neurological function and relieve pain in these patients. En bloc excision is an effective treatment strategy to improve survival and prevent local recurrence. Management of spinal SS should be under the instruction of a multidisciplinary team.

## Background

Primary bone tumours of the spine are relatively rare, comprising only 10% or less of all bone tumours [1]. Synovial sarcoma (SS) is a malignancy that accounts for 6–9% of all soft tissue sarcomas (STSs) and is primarily seen in adolescents and young adults [2]. Two thirds of all SS cases are located in extremities, while less than 5% are found in the spine [3]. It can arise from the osseous and paravertebral soft tissues and even metastasize from other sites [4, 5]. With the enlargement of a SS, the mass can result in pain and other symptoms [6, 7]. Spinal instability, vertebral collapse and neurologic deficits may occur from the destruction of vertebrae structure and compression of nerve roots and the spinal cord [8, 9].

Surgical resection with negative margins is the initial treatment for SS [10]. However, sometimes a complete resection of lesions in the spine cannot be achieved due to the complex anatomical structure and the involved critical neurovascular tissue [11]. For unresectable SS, preoperative chemotherapy or radiotherapy could help downstage large, high-grade tumours and enable effective surgical resection. Adjuvant radiotherapy and/or chemotherapy are also recommended for patients with a microscopically positive margin or with high-grade histological types [12].

Due to its rarity, only a few SS cases involving the spine have been reported, most without follow-up or with a short follow-up period. Additionally, it is commonly accepted that the diagnosis and treatment of spinal SS is challenging [13, 14]. Here, we summarize the clinical features, treatments and outcomes of 16 patients with spinal SS admitted in our institution and present our experience.

## Methods

### Patient samples

This study was approved by the institutional review board of the Changzheng Hospital of the Second Military Medical University. We retrospectively reviewed the clinical and follow-up data of confirmed spinal SS patients who were surgically treated in our institution from August 2008 to May 2017. General information, radiological findings, pre- and post-operative status, treatment strategies, operation details, complications and pathological findings were collected. A total of 16 patients with spinal SS were identified, including 11 men and 5 women, ranging in age from 12 to 68 years (median 33). Radiologic examinations, including a plain radiograph, computed tomography (CT) scan and magnetic resonance imaging (MRI), were used for preoperative diagnosis and disease evaluation. Histological diagnosis was confirmed by needle biopsy or open biopsy. Of the 16 cases, 5 occurred in the cervical spine, 4 in the thoracic spine, 3 in the lumbar spine and 4 in the sacrum.

### Clinical features

The clinical presentations of all patients were collected. Features of radiologic examinations conducted before surgery were analysed. The Weinstein-Boriani-Biagnini (WBB) system was used for surgical staging [15]. Pre- and postoperative Frankel grading was carried out to assess the patient’s neurologic status. The visual analogue scale (VAS) for assessing pain and the Karnofsky Performance Status (KPS) Scale were used before and 3 months after the operation. All patients were routinely followed up 1 and 3 months after the surgery and then at a 3-month interval for the first 1 year and then once every year thereafter. Patients’ conditions were all confirmed by telephone calls at the end of the study.

### Statistical analysis

Continuous variables are expressed as the mean ± standard deviation (SD). The Wilcoxon signed rank test was used to compare the Frankel grades and VAS and KPS scores pre- and post-operation. The Kaplan-Meier method was used to estimate survival. Overall survival (OS) was used as the primary end point and was defined as the interval between the first diagnosis and either death or the date of last follow-up. *P* < 0.05 was considered as statistically significant.

## Results

### General information

Various symptoms were recorded in these 16 spinal SS patients. Pain (15/16, 93.75%), numbness and weakness of extremities (12/16, 75.0%); limited motion of the spine (10/16, 62.5%); palpable mass (11/16, 68.75%); and disturbance of urination or defecation functions (3/16, 18.75%) were commonly observed. The mean preoperative duration of symptoms was 11.8 ± 11.8 months (median 7, range 1–42). The mean preoperative VAS and KPS scores were 5.88 ± 1.65 (median 6, range 3–9) and 45.3 ± 15.0 (median 40, range 20–70), respectively, with Frankel scores ranging from A to E (Table 1). In all 16 patients, 8 had primary lesions, while 6 had local recurrent lesions and 2 had metastatic lesions from the extremities with prior surgical treatments conducted at other institutes (Table 2).

**Table 1:** Demographic data of 16 spinal SS patients

	Spinal SS, *n* = 16
Sex, M/F	11/5
Age, ≤ 30/> 30	8/8
Preoperative KPS, < 60/60–80/≥ 80	12/4/0
Preoperative Frankel scores, A–C/D–E	7/9
Preoperative VAS scores, ≤ 5/> 5	6/10
Tumour size (mean ± SD, cm)	7.57 ± 3.33
Recurrent or metastasis lesions, no/yes	8/8
Preoperative chemotherapy, no/yes	13/3
Operation time (mean ± SD, min)	292.9 ± 121.2
Intraoperative blood loss (mean ± SD, ml)	1547 ± 1152.8
Complication, no/yes	15/1
Postoperative recurrence, no/yes	12/4
Postoperative distant metastasis, no/yes	13/3
Adjuvant chemotherapy, no/yes	10/6
Adjuvant radiotherapy, no/yes	10/6

*SS* synovial sarcoma, *VAS* visual analogue scale, *KPS* Karnofsky Performance Status, *SD* standard deviation, *F* female, *M* male

**Table 2:** General information, treatment, and outcome of 16 patients involved

No. case	Age, years	Sex	Tumor Size, cm	Tumor sites	Recurrent case	Complications	WBB Staging	Pre-FS	Post-FS	Pre-Vas	Post-Vas	Pre-KPS	Post-KPS	Operation duration, minutes	Blood Loss, ml	Surgical approach	Surgical resection	Pre-CT	Pre-RT	Post-CT	Post-RT	Metastasis, months	Local recurrence, months	Diagnosis to event, months	Last Status
1	15	F	10*5	C3-C5	Y	N	A-B/1-3	E	E	4	1	70	90	140	600	pst	En bloc	N	N	N	N	N	N	74	NED
2	59	M	5.7*4	right S	N	N	A-D/9-12	D	E	5	1	60	80	300	1000	pst	En bloc	N	N	Y	Y	bilateral lungs, 8	N	22	AWD
3	59	M	7*8	left S	N	wound infection	A-F/1-6	C	D	8	8	30	30	330	2800	pst	Piecemeal	N	N	N	N	N	3	4	DOD
4	17	M	9*5	L3-S1	Y	N	A-E/7-9	C	D	5	4	40	60	220	1200	pst	Piecemeal	N	Y	Y	Y	right lung, 24	10	47	DOD
5	53	M	14*10	L5, right S C6-T2 C6-T2	N	N	A-E/7-12	C	D	6	6	40	40	330	4500	pst	En bloc	N	N	N	N	N	N	30	NED
6	12	M	3*3 6*5		Y Y	N N	A/1-3 11-12 A-B/1-3 11-12	E D	E D	6 6	3 6	70 60	80 60	180	200	pst	Piecemeal	Y	N	N	N	N	10	/	/
7	53	M	5*4	C7	N	N	A-C F/7-12	E	E	3	1	60	80	300	300	pst	Piecemeal	Y	N	N	N	N	8	34	DOD
8	18	M	5*4	T12-L1	Y	N	A-C F/1-3 8-12	D	E	6	2	50	80	360	3000	ant+pst	En bloc	N	N	Y	Y	N	N	11	NED
9	68	M	7*5	T11	N	N	A-C/1-4	D	E	6	4	30	50	500	2000	pst	Piecemeal	N	Y	Y	Y	N	N	33	NED
10	20	F	4*4	C3-C5	Y	N	A-F/4-12	D	D	8	3	30	80	240	400	pst	Piecemeal	N	N	N	N	N	N	6	DOD
11	23	M	8*5	right S	N	N	A-E/7-12	D	E	6	1	50	80	240	1200	ant	Piecemeal	N	N	N	N	N	N	21	NED
12	26	M	13*10	T1	Y	T1 compression fracture ^a^	A-F/7-12	B	D	8	3	30	50	140	300	pst	Piecemeal	N	N	Y	Y	N	N	54	NED
13	13	F	5*4.5	C6-T1	N	N	A-F/1-5 11-12	C	E	4	1	40	80	510	1600	ant+pst	Piecemeal	Y	Y	N	N	N	N	87	DOD
14	58	F	4*3	C5-C7	Y	N	A-F/5-12	A	C	9	5	20	40	480	1200	ant+pst	Piecemeal	N	N	Y	Y	N	N	60	NED
15	40	F	10*5	L3-L4	Y	N	A-F/1-3 9-12	C	D	6	6	40	40	360	2200	ant+pst	Piecemeal	N	Y	N	N	N	N	39	DOD
16	53	M	12*7	S	N	N	A-E/1-4 10-12	D	D	4	4	50	50	170	2200	pst	Piecemeal	Y	N	N	N	N	5	27	DOD
														180	1600	pst	Piecemeal	N	N	N	N	subcutaneous tissue with skin ulceration, 18	N	25	DOD

*F* female, *M* male, *Y* yes, *N* no, *FS* Frankel grading score, *VAS* visual analogue scale, *KPS* =Karnofsky Performance Status, *WBB* Weinstein-Boriani-Biagnini system, *ant* anterior approach, *pst* posterior approach, *NED* no evidence of disease, *AWD* alive with disease, *DOD* died of disease, *CT* chemotherapy, *RT* radiotherapy

^a^Disease related complications: T1 pathologic fracture found pre-operatively in our institute

### Radiologic evaluation

**Fig. 1:**
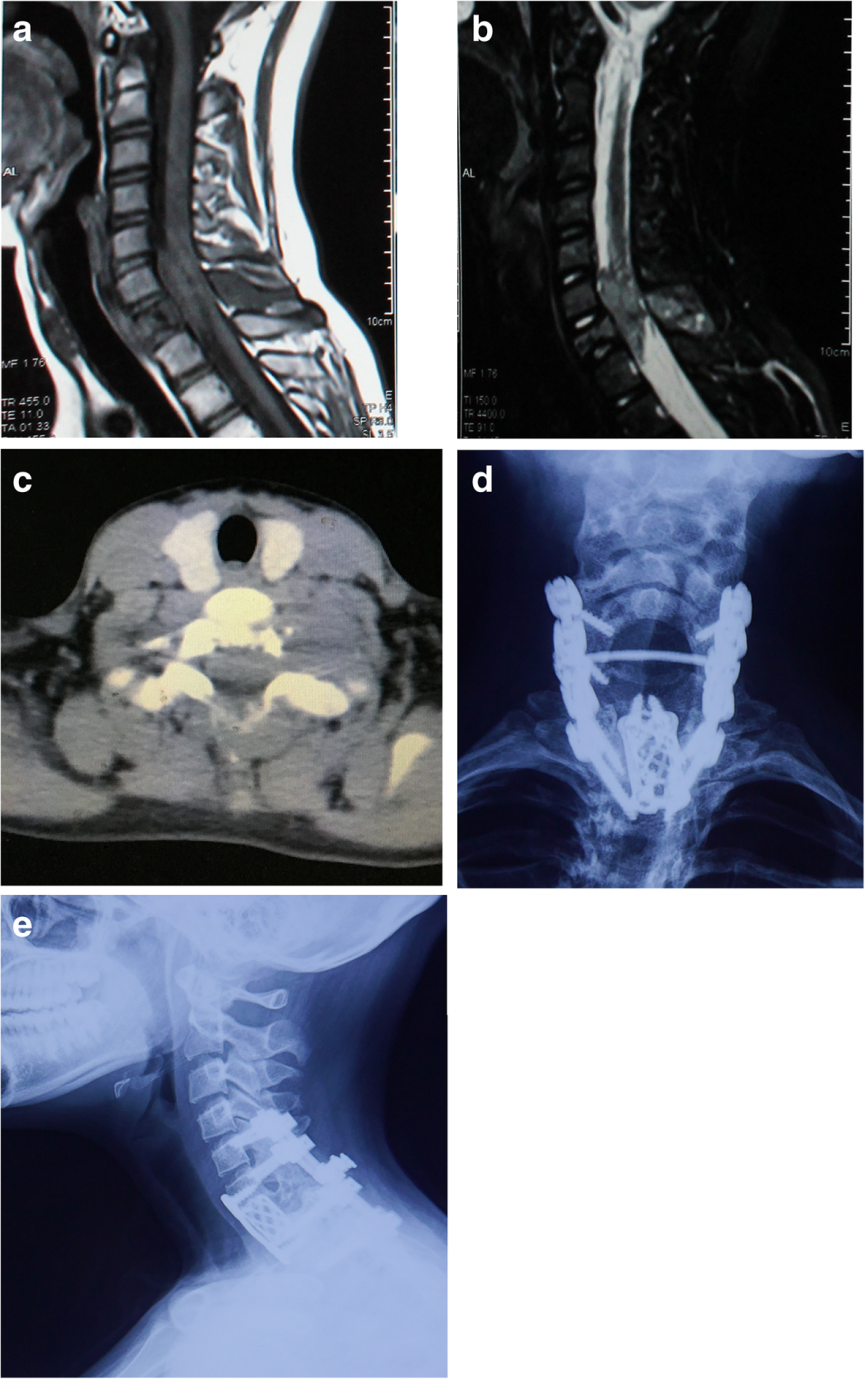
Patient no. 13: MRI showing low signals in T1- (**a**) and heterogeneous signals in T2 (**b**)-weighted images of a soft tissue mass in the C7 vertebral body and appendix, with spinal cord compression from C6 to T1. CT images (**c**) showing a destructive soft tissue mass and osteolytic lesion in the C7 vertebral body and appendix. Cervical vertebra anterior and posterior (AP) (**d**) and lateral (LAT) (**e**) X-rays at the follow-up 52 months after surgery, demonstrating the maintenance of the instruments and spinal stability

**Fig. 2:**
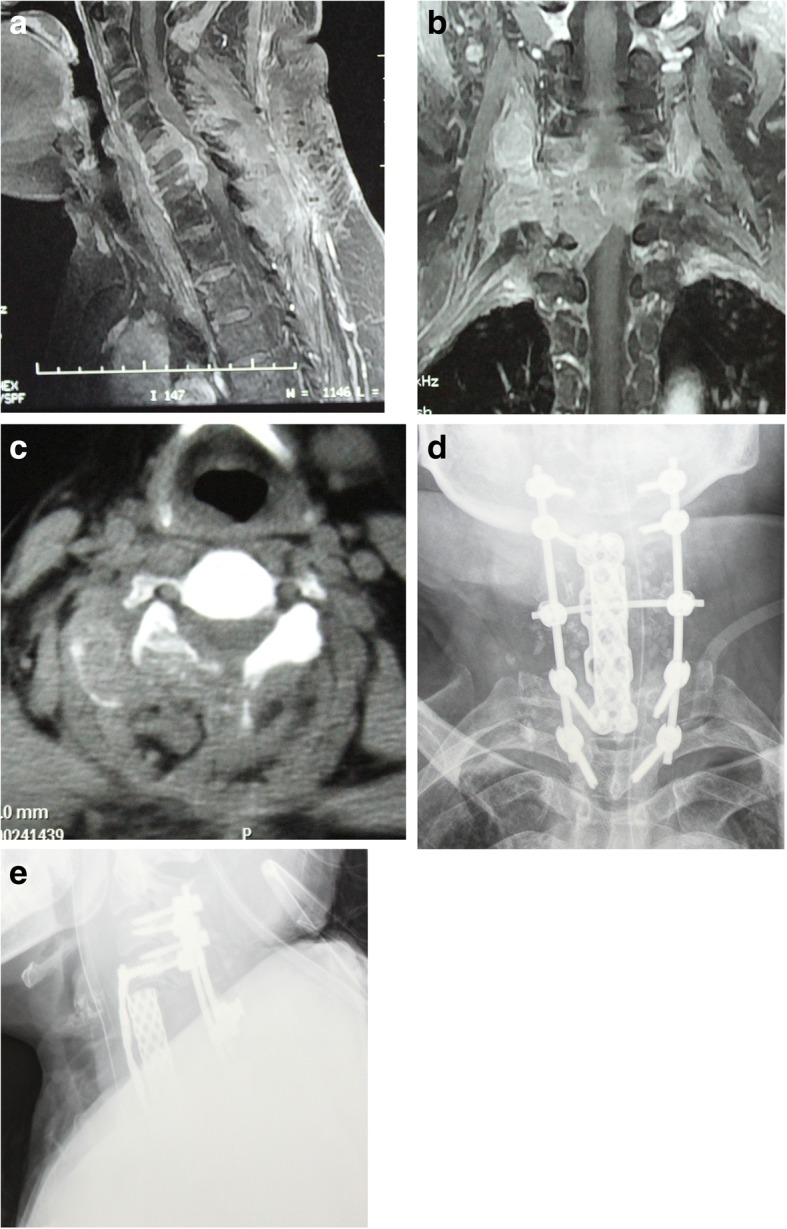
Patient no. 14: MRI showing a vertebral lesion with a paraspinal mass from C5 to C7 (**a**, **b**). CT images (**c**) showing an osteolytic lesion with calcification. AP (**d**) and LAT (**e**) X-rays after surgery, showing anterior vertebral reconstruction and posterior internal fixation

Plain radiographs showed an osteolytic lesion with a well-defined soft tissue mass in 10 out of 16 patients. Based on the preoperative radiologic imaging, the average tumour size was 7.57 ± 3.33 cm. Collapse of the vertebral body was seen in one patient with a pathological fracture of T1. CT images showed osteolytic lesions in vertebral body and/or appendix (16/16), with paravertebral or epidural soft tissue masses (12/16). Most tumours were well demarcated, and tumour calcification was found in three patients. MRI revealed heterogeneous signals in T1- and T2-weighted images of the vertebral and paravertebral lesions (Figs. 1 and 2).

### Surgical intervention

All patients were surgically treated. The surgical approach and the instrumentation method were tailored for each patient. Four en bloc resections and 12 piecemeal resections were performed in the 16 spinal patients, with an average procedure time of 292.9 ± 121.2 (median 300, range 140–510) min and blood loss of 1547 ± 1152.8 (median 1200, range 200–4500) ml (Table 1). All resected specimens were proven with a negative margin by pathological examination. Intraoperatively, the surgical field was immersed with oxaliplatin. Spinal stability and balance were restored in accordance with the resection extension. Artificial vertebral bodies, titanium mesh cages and anterior titanium plates were applied to reconstruct the bony defects. Pedicle screw systems and lateral mass screw systems were used for posterior internal fixation (Figs. 1 and 2). A significant amelioration of Frankel scores was detected postoperatively (*P* = 0.002). VAS (*P* = 0.002) and KPS (*P* = 0.002) scores were also significantly improved after operation in all patients.

### Adjuvant treatment

Radiotherapy and chemotherapy were used before and/or after operation based on the tumour size, surgical findings and the general status of a patient. Because of recurrent diseases and the need to facilitate surgical resection, a total of three cases received preoperative chemotherapy. Six patients had both adjuvant chemotherapy and radiotherapy. Gamma knife was performed on the pulmonary metastasis of one patient.

### Follow-up

The average time of follow-up for all 16 patients was 35.9 ± 23.5 (median 31.5, range 4–87) months. The 1-, 3- and 5-year OS rates were 87.5%, 61.4% and 40.9%, respectively. Four of the 16 patients (25.0%) developed local recurrence 3–10 months after surgery in our institution and one of them underwent another surgical resection. All four of these patients, three of whom had a history of recurrent disease, received piecemeal resection. Three of the 16 patients (18.75%) developed distant metastasis 8–24 months after the operation, including two lung metastases and one metastasis of the subcutaneous tissue with ulceration. Eight of the 16 patients (50.00%) died during the follow-up period, with a mean survival of 33.6 ± 26.2 (median 30.5, range 4–87) months. All eight of these patients received piecemeal resection, and the causes of death included local or distant recurrence or multiple organ failure after the operation. One (6.25%) patient was alive with the disease (AWD) at last follow-up, and the other seven (43.75%) were living without evidence of disease (NED). Postoperative complications, including wound dehiscence and fat liquefaction, were observed in one patient (Table 2).

## Discussion

SS is a rare and aggressive malignancy, with a predominance in adolescents and young adults [2]. Similarly, 8 of the 16 (50.0%) patients in our study were younger than 30. This type of tumour has the potential for metastasis, with the lungs being the most susceptible site [16]. In our series, there were two patients who had a lung metastasis. One patient experienced a subcutaneous tissue metastasis, which is uncommon and has only previously been reported in prior literature twice [17]. SS occurs in the extremities, most commonly near the joints, and the rarity of spinal SS limits the sample size of studies conducted on this type of cancer [18]. Most of the available knowledge on the treatment and prognosis of spinal SS comes from sporadic cases reported in previous literature (Table 3). As far as we can tell, this is the largest conducted case series that analyses the clinical features and outcomes of these patients.

**Table 3:** Review of spinal SS reported in the literature by year since 2008

Literature by year	Number of cases	Age, years	Sex	Tumour sites	Treatment	Follow-up
Subramanian et al. 2018 [19]	1	47	F	T7-8	Laminectomy and total excision of the tumour followed by posterior fusion, adjuvant radiation and chemotherapy	Improvement in the neurological status and remained disease free at 6 months follow-up
Guo et al. 2016 [21]	1	10	M	T9-10	Laminotomy and total excision of the tumour, adjuvant radiation therapy and ifosfamide chemotherapy	No symptoms recurred 6 months after surgery
Yang et al. 2016 [32]	1	20	M	C2	Subtotal resection of the tumour	In situ recurrence after 6 months, patient succumbed to the disease 1 month later
Chen et al. 2016 [34]	1	20	F	T12-L2	A posterior tumour resection with decompression and postoperative radiotherapy	/
Cao et al. 2014 [33]	1	26	M	T7	T7 en bloc resection followed by radiation therapy and chemotherapy	Low back pain in 1 year after surgery
Kim et al. 2014 [6]	1	29	M	C2-3	Marginal resection followed by adjuvant radiation therapy	Disease free for 2 years
Garg et al. 2013 [24]	1	45	F	Lumbosacral paraspinal area	Wide local excision of the lump with lateral intercostal artery-based rotational flap reconstruction	/
Peia et al. 2013 [22]	1	7	F	L4-5(L5 nerve root)	Bone erosion extended and the infiltrated root resected, followed by chemotherapy	No evidence of disease after 5 years
Kim et al. 2013 [5]	1	17	M	C3	Tumour resection of C3, laminectomy of C4, partial laminectomy of C2 and posterior instrumentation of C2-C6; anterior cervical fusion of C2-C5; postoperative chemotherapy	/
Yonezawa et al. 2012 [36]	1	23	F	L3-4(cauda equina)	L2-4 left hemilaminectomy, tumour totally resected and adjuvant local radiation therapy	Free of local recurrence and metastasis 5.5 years after surgery
Naphade et al. 2011 [37]	1	14	M	C6-7	A well-defined epidural mass lesion completely excised	No signs of local recurrence or metastasis at 6 months post surgery
Zairi et al. 2011 [29]	1	36	M	C1-2	Complete resection and radiotherapeutic adjuvant treatment	Local recurrence; multiple lung metastases occurred in 6-year follow-up and died of disease
Puffer et al. 2011 [18]	3	59	F	T4-6	(1) T3-5 laminectomy with debulking of tumour, (2) en bloc resection of T4-7 with a posterior instrumented spinal fusion from T1-L1 and (3) radiation therapy and chemotherapy	No evidence of tumour recurrence or metastases at 67 months from the final resection
		54	F	Paraspinal mass centred around T10	T12-L1 laminectomy and biopsy	Died of disease 4 months later
		32	F	T5-6	Paraspinal tumour removed, dura incised and nerve root resected with negative margins; gross residual tumour in the T6 transverse process resected; followed by chemotherapy	No tumour recurrence at 6-month follow-up; suspicious lung metastases
Foreman et al. 2011 [23]	1	29	M	C3-T1	Subtotal resection, postoperative radiation therapy and chemotherapy	6 years post resection without recurrence of the tumour
Liu et al. 2010 [35]	1	12	M	S2 and below	Tumour en bloc excision and postoperative radiation therapy	Recurrence at 6 months after surgery and died of disease 21 months after diagnosis
Arnold et al. 2010 [30]	1	26	F	C2-5	C2-3 laminectomy, posterior C2 corpectomy with occipital-C7 fixation and fusion, palliative chemoradiation	Died 6 months postoperatively of disease progression
Ravnik et al. 2009 [8]	1	32	M	T12-L2	Laminectomy with epidural mass removal; debulking surgery; following chemoradiation	Local recurrence after 12-month follow-up
Koehler et al. 2009 [4]	1	60	M	T7-10	Right-sided thoracotomy, followed by radiation therapy	No recurrence in 9-month follow-up
Barus et al.2009 [10]	1	14	F	L2-5	Marginal resection with anticipated postoperative chemotherapy and radiation	No evidence of local recurrence or distinct disease in 5 years and 9 months after surgery
Scollato et al. 2008 [31]	1	59	M	C3-5	Longitudinal myelotomy	Died of lung and hepatic metastases 3 months later

*F* female, *M* male

Progressive chronic pain and a palpable mass are the common symptoms of SS [2, 14, 19]. In a previous report, the duration between the onset of symptoms and start of treatment could be longer than 10 years [20]. For spinal patients, numbness or weakness of the extremities and urinary or bowel dysfunction caused by nerve root irritation or spinal cord compression are commonly seen [21–23]. In our series, 81.25% patients had varying degrees of neurological deficits and almost all patients had pain in tumour sites. One patient was admitted to our institution with acute lower extremity paralysis due to a compression fracture of the T1 vertebral body. His motor and sensory functions were improved significantly after surgery. Calcification seen in plain radiographs and CT scans is thought to be one of the features of SS in approximately 30% of patients [18, 24]. In our series, the calcification feature was seen in three (18.75%) patients. On MRI, most SSs have variable and heterogeneous signals in T1- or T2-weighted images [5]. Radiologic examinations are applied to detect the tumour but are difficult to use in making a diagnosis. No definitive characteristics have been seen to distinguish SS from other diseases like nerve sheath tumours, Ewing sarcoma, chondrosarcoma and other unusual STSs.

Spinal SS has a relatively poor prognosis, with a high died of disease (DOD) risk (50.00%) and a low NED (43.75%) rate, both of which are also documented in previous studies [7, 13, 16, 25]. A total of four postoperative local recurrences and three distant metastases were detected. It has been documented that most spinal SS patients die within 3 years [18], while the 5-year OS rate of all-site SS is 25–76% [7, 26]. In the current study, the 5-year OS was 40.9%, which is consistent with the published data. Sar et al. [27] reported a patient with primary SS of the sacrum who died of this disease 94 months after surgery, which is the longest survival time in literature. In our study, the longest OS was seen in a thoracic spinal SS patient, who had an 87-month survival. There are several possible reasons of the unfavourable prognosis of spinal SS. First, nerve compression can cause neurological deficits, which impacts the quality of life of these patients. Heavy blood loss and long surgical duration also affect the general status of these patients. Second, due to the complex structures of the vertebrae and surrounding tissue, sometimes en bloc excision is hard to achieve, and potential tumour residue might occur. In our study, no local recurrences were seen in the four patients who received an en bloc excision, and they seem to have a relatively good prognosis. Therefore, any tumour residue might increase the risk of postoperative recurrence. On the other hand, the special anatomic site of spinal lesions might help refine the application and dosage of radiotherapy.

Management of spinal SS should follow the instruction of a multidisciplinary team, and surgical resection serves as the first choice, if wide excision with clear margins is possible [28–31]. Subtotal resection of the tumour might be followed shortly by a local recurrence [32]. En bloc resection is important in spinal tumours to minimize the tumour residue. Kim et al. [6] reported a C2 SS removed with negative margins, and Cao et al. [33] reported another case who underwent a T7 en bloc resection. Both patients survived without recurrence in their 2- and 1-year follow-up, respectively. In our series, a total of four patients, including two with cervical lesions, one with lumbar lesions and one with sacral lesions, received en bloc resections conducted as one-stage surgeries, and they all had favourable prognoses post-surgery. None of the four patients developed local recurrence or died of this disease during the follow-up from 11 to 74 months. A two-stage surgery with the tumour removed with negative margins may also achieve satisfying local control. Puffer et al. [18] reported a case of thoracic dumbbell SS. The tumour was grossly debulked in the first stage, and en bloc resection of the T4, 5, 6 and 7 vertebrae was performed as a second-stage operation. No evidence of tumour recurrence was observed at 67 months from the final resection. For complete tumour resection, sometimes nerve roots or vessels must be sacrificed. In a case [22] of a primitive intraneural SS of the L5 nerve root, the infiltrated root was resected for complete removal of the residual lesion. A slight sensory and motor deficit in the left leg persisted during the 5-year follow-up. The decision should be made carefully, and patients should be informed, especially when cervical, lumbar and sacral nerve roots and vertebral arteries are entrapped and violated. In our series, in nine patients, including three with en bloc resection, the nerve roots were sacrificed.

Adjuvant therapies are beneficial for large, deep and high-grade STSs [28]. Six patients with an advanced tumour stage and relatively stable general status had chemotherapy and local radiotherapy following the operation. Of these six patients, one was DOD, one was AWD and four were alive with NED at their last follow-up. The relatively better outcomes for these patients might be affected by their better general condition. Most previously reported cases used adjuvant chemotherapy and/or radiation therapy [34–36]. Naphade et al. [37] reported a case of cervical SS where the tumour was completely excised without adjuvant treatment, and no signs of local recurrence or metastasis were detected at 6 months after surgery. There is no consensus about the role of adjuvant and preoperative chemotherapy in SS [28]. A previous study demonstrated that SS tended to have higher chemosensitivity compared to other STSs [38]. Eilber et al. [39] showed that ifosfamide-based chemotherapy offered a survival benefit to adult patients with primary extremity SS. However, Italiano et al. [12] suggested that preoperative or adjuvant chemotherapy did not improve the prognosis. Based on our study and experience, well-planned, wide surgical excision should be the cornerstone of treatment for spinal SS and the main factor indicating their prognosis. Molecular and cellular abnormalities of SS implied new therapeutic targets, and receptor tyrosine kinase inhibitors like cediranib and bevacizumab have shown promising results [40–42].

## Conclusion

We presented a case series of 16 spinal SS patients, and the features of spinal SS were roughly depicted. Spinal SS has a relatively poor prognosis. Surgical management with en bloc excision is demanding yet the most effective treatment strategy to improve outcomes. Management of spinal SS should be under the instruction of a multidisciplinary team. Therefore, a multi-centred, prospective study with a large cohort is required to further investigate the therapeutic strategy for spinal SS.

## Abbreviations

AWD: Alive with the disease
CT: Computed tomography
DOD: Died of disease
KPS: Karnofsky Performance Status
MRI: Magnetic resonance imaging
NED: No evidence of disease
OS: Overall survival
SS: Synovial sarcoma
STS: Soft tissue sarcomas
VAS: Visual analogue scale
WBB: Weinstein-Boriani-Biagnini
